# Supplementary material for: How Quotation Types Shape Classic Novel Reading in Chinese: A Comparison Between Human Eye-Movements and Large Language Models
Source: Behav Sci (Basel). 2025 Nov 30;15(12):1650. doi: 10.3390/bs15121650 (PMC12729666; doi:10.3390/bs15121650)
Supplement: Supplementary file 1 [file behavsci-15-01650-s001.zip › behavsci-3914277-supplementary.pdf]

Supplementary Materials

Table S1. The statistical results of the finally fitted linear mixed-model analyses of the eye-movement measures.

eye-movement	ROI	Congruency		Quotation type		Congruency× Quotation type	
		χ^2	p	χ^2	p	χ^2	p
FFD	ROI 1	1.673	.196	2.876	.237	7.972	.019
GD	ROI 1	0.983	.321	7.604	.022	4.395	.111
TRT	ROI 1	30.593	<.001	26.311	<.001	4.645	.098
RPD	ROI 1	10.793	.001	13.095	.001	3.347	.188
FFD	ROI 2	3.436	.064	1.669	.434	2.10	.350
GD	ROI 2	1.098	.295	3.344	.188	2.596	.273
TRT	ROI 2	4.097	.043	11.04	.004	1.191	.551
RPD	ROI 2	7.741	.005	13.439	.001	2.811	.245

Table S2. The statistical results of the finally fitted linear mixed-model analyses of surprisal and entropy derived from LLM metrics.

LLM metrics	ROI	Congruency		Quotation type		Congruency× Quotation type	
		χ^2	p	χ^2	p	χ^2	p
surprisal	Critical	62.671	<.001	0.015	0.993	0.026	.987
	Post-1	0.008	.929	62.61	<0.001	0.775	.679
	Post-2	0.062	.803	135.16	<0.001	0.093	.955
	Post-3	0.159	.690	49.199	<0.001	0.008	.996
entropy	Critical	26.615	< .001	0.022	0.989	0.020	.989
	Post-1	1.200	.273	4.885	0.087	0.526	.769
	Post-2	1.093	.296	7.305	0.026	0.256	.880
	Post-3	0.746	.388	5.173	0.075	0.003	.998
